# Amyloidosis Masquerading as Alcohol-Related Cirrhosis

**DOI:** 10.7759/cureus.8976

**Published:** 2020-07-02

**Authors:** Navroop Nagra, Blaire Burman, Christopher R Gault, Russell Dorer, Asma Siddique

**Affiliations:** 1 Gastroenterology, Virginia Mason Medical Center, Seattle, USA; 2 Hepatology, Virginia Mason Medical Center, Seattle, USA; 3 Pathology, Virginia Mason Medical Center, Seattle, USA

**Keywords:** amyloidosis, ascites, alcohol, hepatic amyloidosis, cardiac amyloidosis, cirrhosis, ascitic fluid analysis, multiple myeloma

## Abstract

Amyloidosis can affect multiple organs, and involvement of the heart is the most common cause of death. Signs and symptoms vary depending upon the organ system affected by amyloid. Liver involvement is often seen, but symptoms are usually mild and nonspecific in isolated hepatic amyloidosis. None of the laboratory markers and imaging is characteristic of this condition; therefore, diagnosis is often delayed. Tissue biopsy is required for definitive diagnosis. Herein, we report a case where the patient’s symptoms had been attributed to alcohol-related cirrhosis; however, further workup ultimately led to a diagnosis of systemic amyloidosis with multi-organ involvement.

## Introduction

Amyloidosis is a systemic disorder characterized by the deposition of an insoluble fibrillar protein in extracellular space [1-3]. Light chain (AL) amyloidosis is caused by plasma cell dyscrasia and usually multiple organs are involved by the time of diagnosis. Cardiac involvement leads to diffuse thickening of myocardium causing arrhythmias, syncope, restrictive cardiomyopathy, and other sign and symptoms of diastolic heart failure, including ascites and peripheral edema [4]. Ascites due to heart failure is characterized by elevated total protein in ascitic fluid (>2.5 g/dL). Hepatic involvement is also common in amyloidosis and mostly causes mild and nonspecific symptoms. Right upper quadrant (RUQ) pain due to hepatomegaly is common; however, severe cholestasis and intractable ascites are exceedingly rare [5]. Prognosis is extremely poor in patients with multi-system involvement with amyloidosis.

## Case presentation

**Figure 1:**
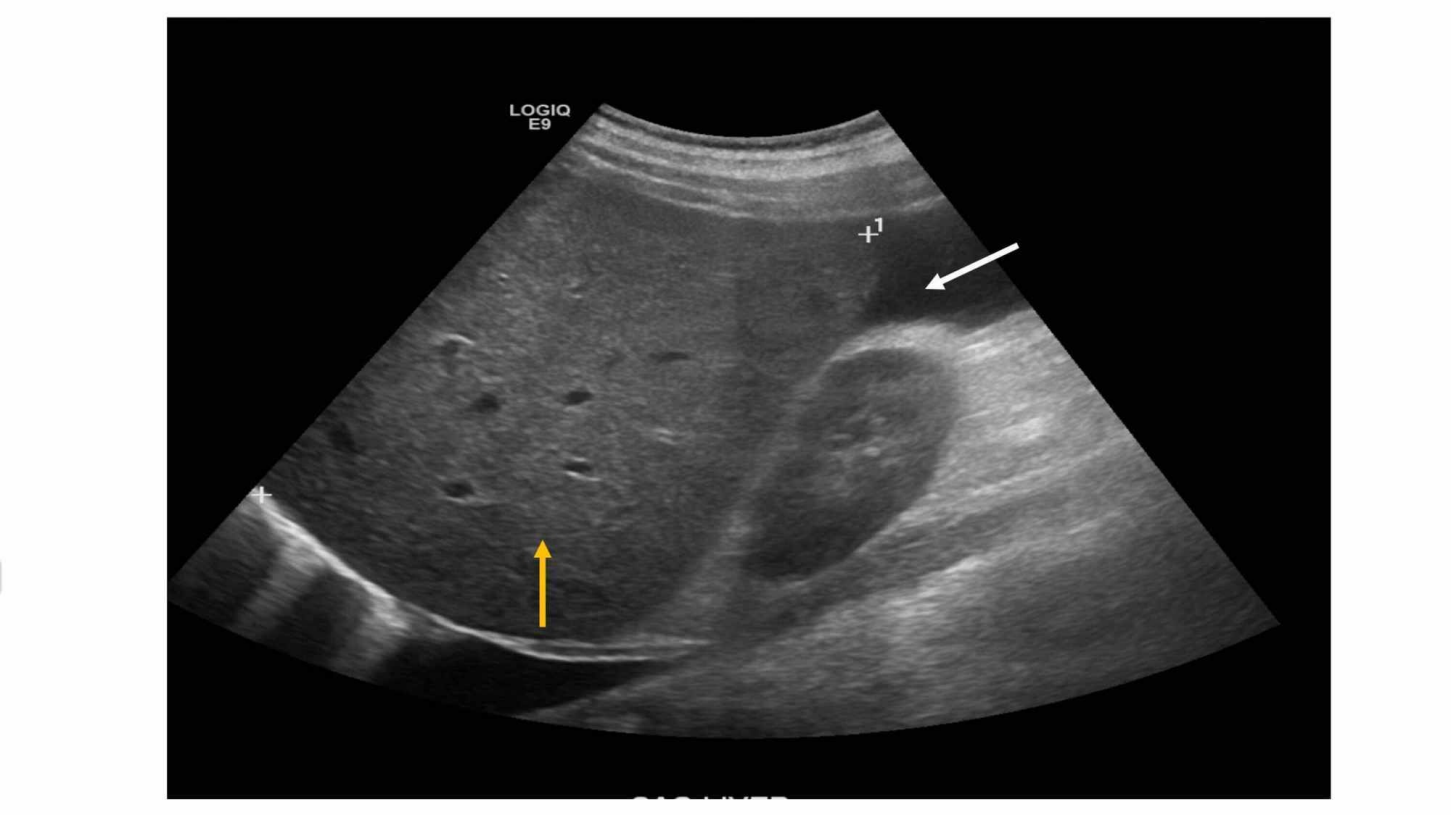
Ultrasound of the abdomen Ultrasound showing hepatomegaly, hepatic steatosis (yellow arrow), and ascites (white arrow).

A 44-year-old man with no significant previous medical history presented to his primary care (PCP) with four-month history of exertional shortness of breath (SOB), abdominal distension, and leg edema. Blood work at his PCP office revealed normal complete blood count (CBC), albumin 4.7 g/dL, bilirubin of 1.5 mg/dL, alkaline phosphatase (ALP) 197 U/L, aspartate transaminase (AST) 60 U/L, and alanine transaminase (ALT) 57 U/L. Hepatitis B and C serologies were negative. Ultrasound (US) of the abdomen reported hepatic steatosis, mild hepatomegaly, and ascites (Figure 1).

**Figure 2:**
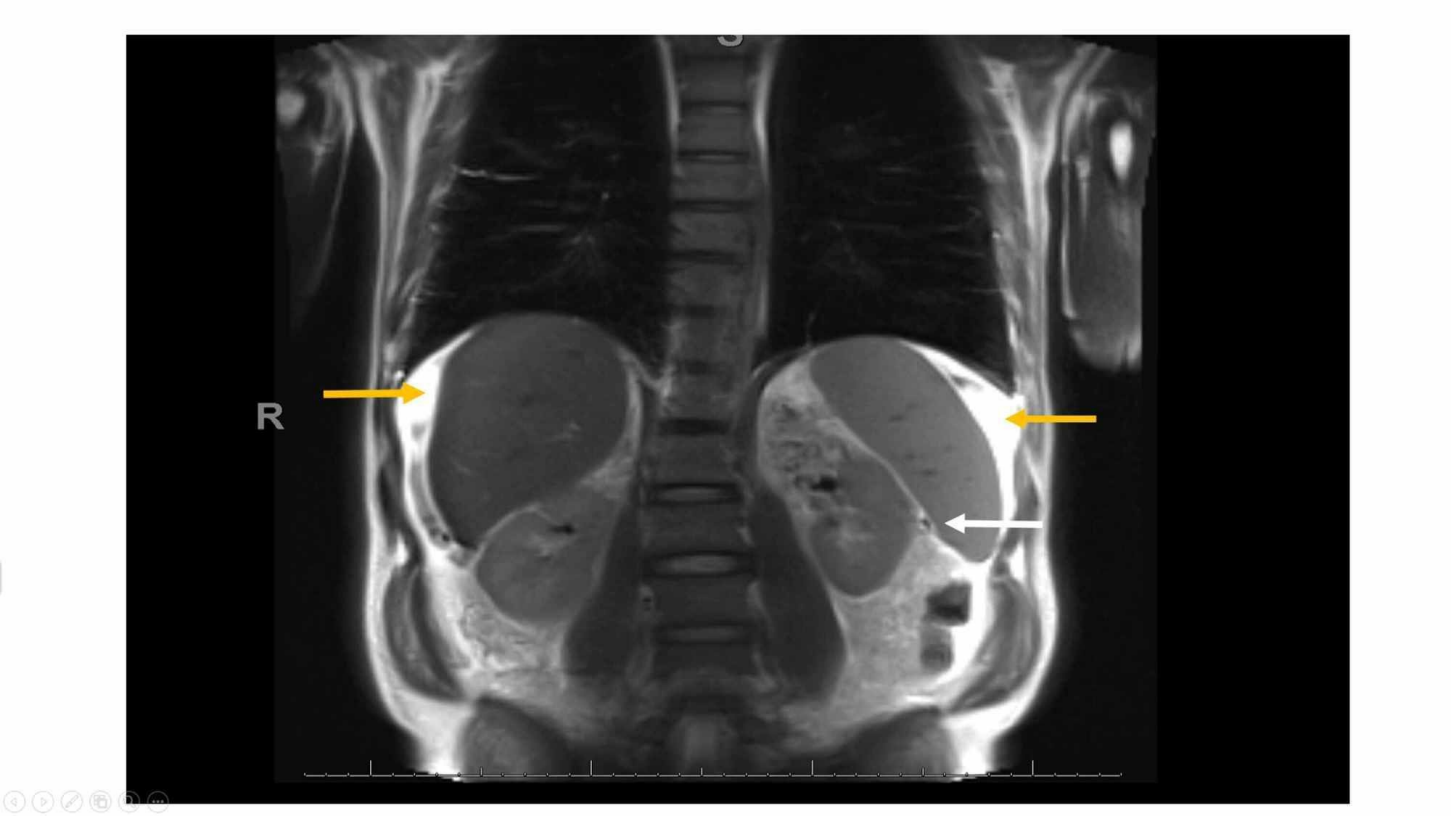
MRI and magnetic resonance cholangiopancreatography of the abdomen MRI showing ascites around liver and spleen (yellow arrows), and splenomegaly (white arrow).

Cardiac evaluation with dobutamine stress echocardiogram revealed no signs of reversible ischemia and normal ejection fraction (EF). He had a long-standing history of alcohol use (on average three to four drinks per day) for around 20 years. His symptoms and laboratory values were attributed to "presumed alcohol-related cirrhosis". He was advised to abstain from alcohol and was started on oral diuretics with some improvement in symptoms. Paracentesis was not performed before starting diuretics as his ascites was mild, and his main complaint was leg edema and SOB. He was referred to hepatology for further evaluation. On his presentation to hepatology clinic, he reported improvement in abdominal distension but worsening leg edema, and new-onset scrotal swelling. Further workup was ordered. His iron panel was not reflective of iron overload and, ceruloplasmin, alpha 1 anti-trypsin, anti-smooth muscle antibody, and immunoglobulin G were all normal. MRI and magnetic resonance cholangiopancreatography (MRCP) of the liver showed small ascites and splenomegaly (Figure 2).

No liver nodularity was seen. Dose of diuretics was increased, and low salt diet was recommended. His symptoms improved transiently. Outpatient upper endoscopy did not show esophageal or gastric varices.

**Figure 3:**
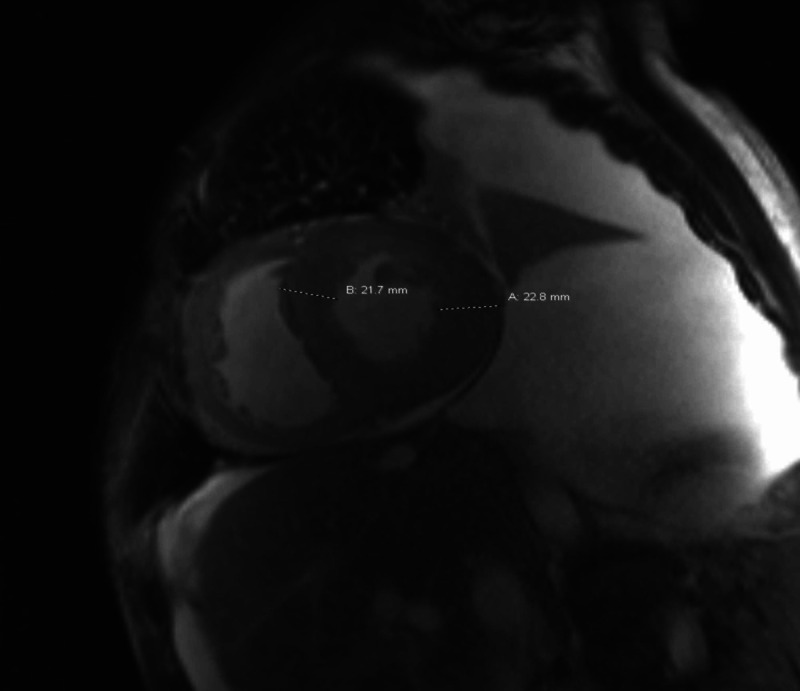
Cardiac MRI Concentric left ventricular myocardial thickening measuring over 20 mm, up to approximately 23 mm in the lateral wall (normal mid cavity thickness is 5-9 mm).

Four months later, he presented to the emergency room (ER) with a worsening SOB, painful abdominal distension, increased scrotal edema, and leg swelling. Relevant blood work showed significant hyponatremia with sodium of 123 mmol/L, creatinine 1.9 mg/dL, AST 70 U/L, ALT 67 U/L, ALP 235 U/L, albumin 4.2 g/dL, and bilirubin 1.9 mg/dL. Electrocardiogram (EKG) showed sinus tachycardia and low voltage in frontal leads. He underwent paracentesis, and ascitic fluid polymorphonuclear neutrophil (PMN) cell count was high at 2,063 cells/mcL, culture was negative, and he was started on ceftriaxone for spontaneous bacterial peritonitis (SBP). Serum ascites albumin gradient (SAAG) was 1.4 g/dL consistent with portal hypertension, and ascitic fluid total protein was high at 3.2 g/dL concerning for cardiac ascites. Cytology showed rare inflammatory cells, acellular proteinaceous material, and no malignant cells. Repeat echocardiogram showed EF of 40%, severe left ventricular hypertrophy (LVH), grade III diastolic dysfunction, moderately reduced right ventricular systolic function, and a "speckled" myocardium consistent with restrictive cardiomyopathy. A cardiac MRI was then performed which revealed findings compatible with a diffusely infiltrative myocardial process most suggestive of cardiac amyloidosis and a decline in EF to 35% (Figure 3).

He was started on intravenous diuretics with significant improvement in anasarca and approximately 30 lbs weight loss. He continued to require frequent paracentesis during his inpatient stay. Ascitic fluid PMNs remained persistently elevated with negative cultures. CT of the abdomen and pelvis with intravenous and oral contrast was performed, which did not show perforation of the gastrointestinal tract. There was no evidence of peritoneal nodularity or carcinomatosis. Secondary peritonitis was ruled out. He continued to stay afebrile, had normal white cell count, and all cultures were negative; therefore, antibiotics were discontinued.

**Figure 4:**
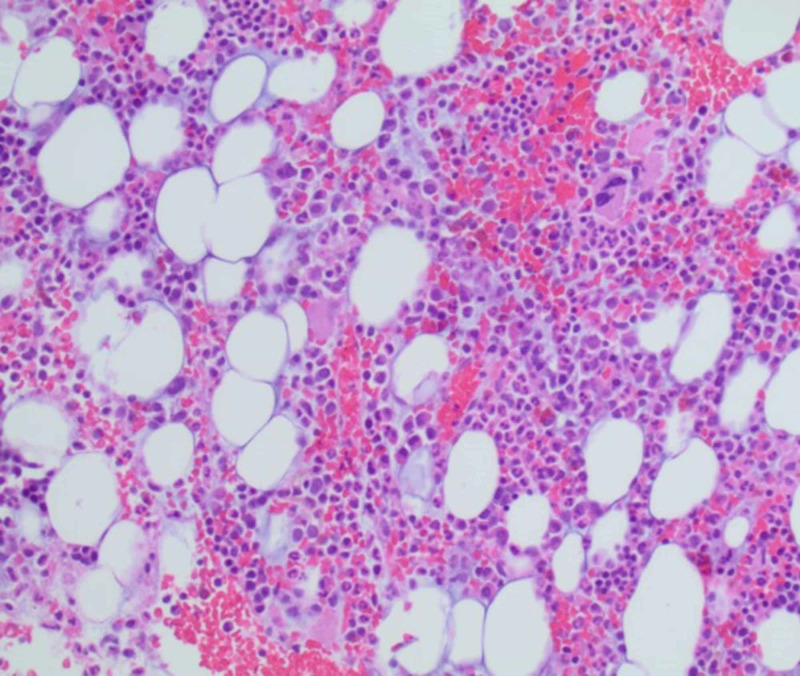
Bone marrow biopsy Mildly hypercellular marrow with trilineage hematopoeisis.

**Figure 5:**
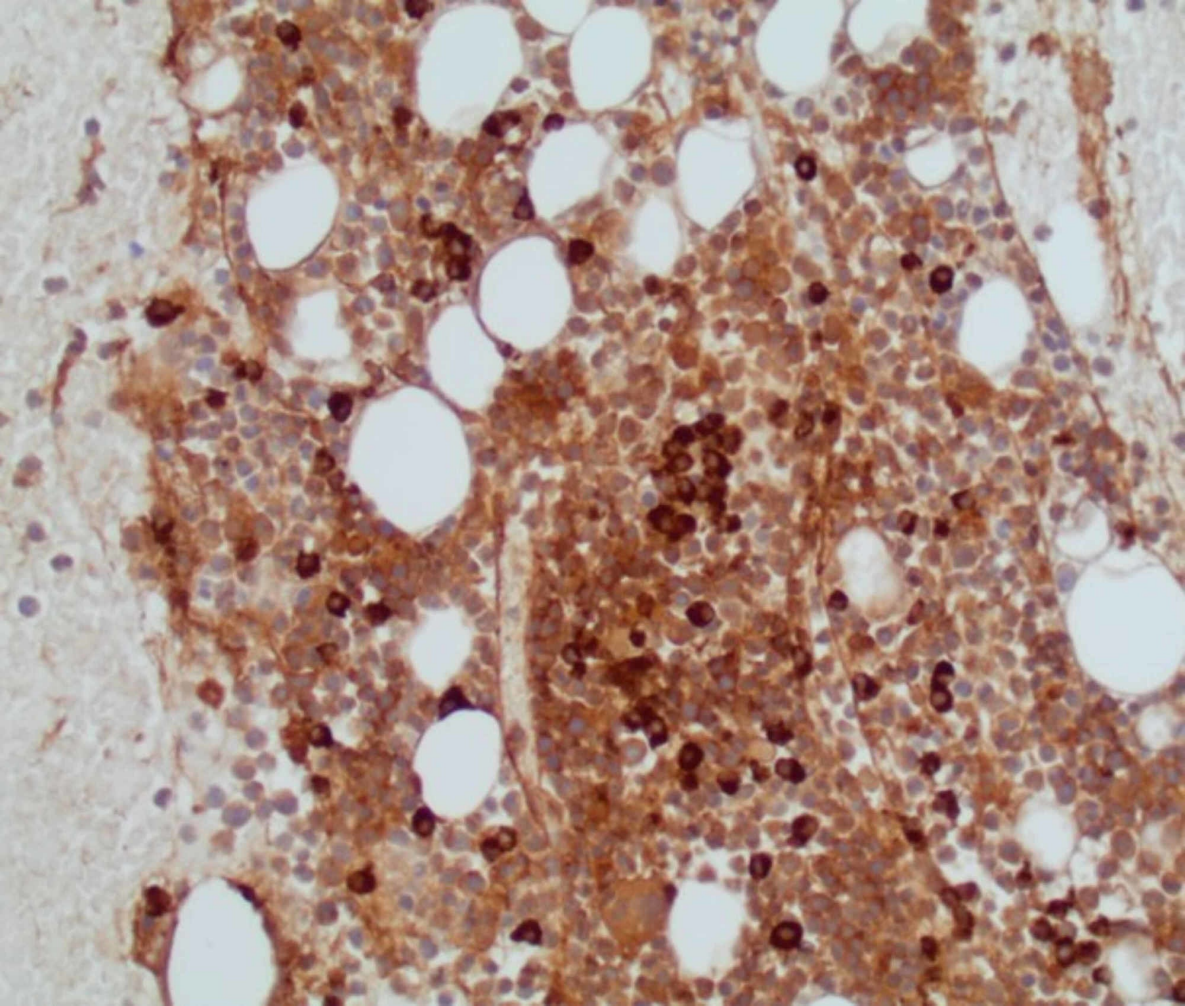
Bone marrow biopsy Stain for lambda light chain showing increased lambda-restricted plasma cells.

Further investigations in the form of serum and urine electrophoresis revealed elevation of lambda light chains in serum and urine at 50.9 mg/dL (0.57-2.63 mg/dL) and 5.85 mg/dL (<0.700 mg/dL), respectively, concerning for AL amyloidosis. This led to a bone marrow biopsy, which showed 15%-20% plasma cells consistent with multiple myeloma (MM) (Figures 4, 5).

**Figure 6:**
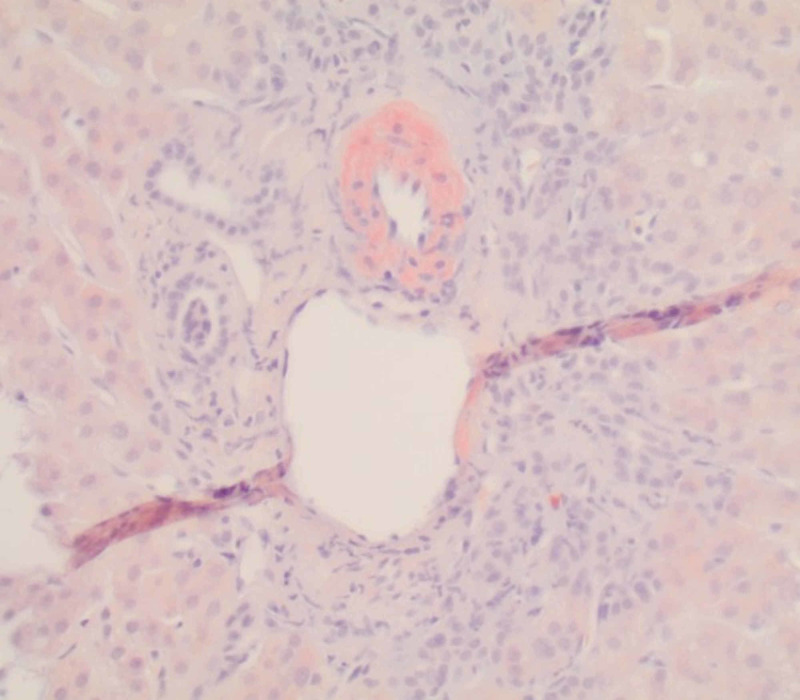
Liver biopsy Congo red stain showing positive staining within the hepatic artery.

**Figure 7:**
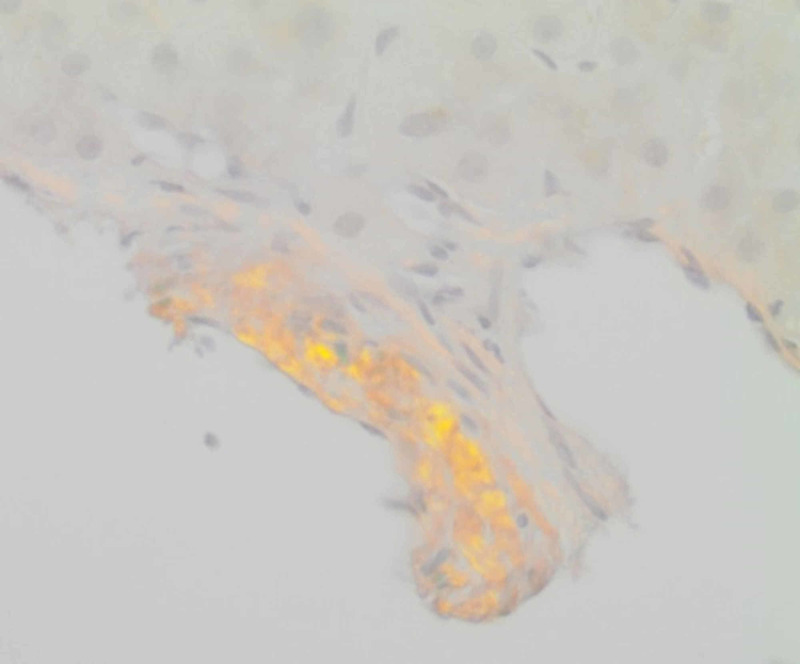
Liver biopsy Congo Red stain under polarized light showing the typical “apple-green birefringence” of amyloid.

Transjugular liver biopsy with portal pressure measurements was performed to rule out cirrhosis due to the rarity of hepatic amyloidosis causing ascites. Hepatic venous pressure gradient was normal at 4 mmHg ruling out portal hypertension. Liver biopsy showed Congo red stain positive for amyloid involving hepatic arteries and a detached fragment of a larger vessel, along with stage III fibrosis (Figures 6, 7).

His final diagnosis was therefore established as MM causing AL amyloidosis with the involvement of heart, liver, and possibly peritoneum. His liver fibrosis was attributed to alcohol and congestive hepatopathy. He was started on bortezomib and dexamethasone for MM and discharged home on diuretics. One week after discharge from the hospital, he presented back to ER with weight gain, abdominal distension, acute kidney injury, and hyperkalemia with potassium of 6.4 mmol/L, and subsequently developed persistent ventricular tachycardia/fibrillation. Unfortunately, despite aggressive resuscitation measures, the patient succumbed to cardiac arrhythmias.

## Discussion

AL amyloidosis, also called primary amyloidosis, is a systemic disorder caused by plasma cell dyscrasia in which there is a deposition of immunoglobulin light chains in extracellular matrix of different organs [6]. It mainly involves the heart, kidneys, liver, gastrointestinal tract, peripheral nerves, and skin.

AL amyloidosis presents most commonly in men after the fourth decade. Some degree of cardiac involvement is seen in 50%-70% of patients with AL amyloidosis [7]. Restrictive cardiomyopathy with predominantly right heart failure is seen, causing peripheral edema, hepatic congestion, ascites, and dyspnea. Echocardiogram is the initial test performed in patients with suspected cardiac amyloidosis. Findings of relative apical sparing of longitudinal strain on echocardiogram is the key feature of cardiac amyloidosis. Cardiac MRI is a better test than echocardiogram with pooled sensitivity and specificity reaching 85% and 92%, respectively, in diagnosing cardiac amyloidosis [8]. LVH is commonly seen and is typically symmetric in AL amyloidosis.

Hepatic involvement is seen in 15% of cases and presents as fatigue and RUQ pain due to hepatomegaly [9]. Elevated liver enzymes with cholestatic predominance and disproportionately high alkaline phosphatase are seen in the majority (86%) of cases [10,11]. Ascites in amyloidosis is mostly due to cardiomyopathy or peritoneal involvement and rarely due to isolated hepatic amyloid [12]. Intractable ascites, portal hypertension, cirrhosis, fulminant hepatic failure, and spontaneous hepatic rupture are exceedingly rare. Tissue biopsy is required to confirm the diagnosis, and classic finding is apple-green birefringence under polarized light with Congo red stain indicative of amyloid deposition.

In our patient, his recurrent ascites was most likely secondary to heart failure, as ascitic fluid revealed high SAAG with high total protein. Interestingly, our patient’s ascitic fluid showed persistently elevated PMN despite antibiotic treatment for presumed SBP. Our assumption is that our patient likely had peritoneal involvement of amyloid based on the presence of acellular proteinaceous material seen on ascitic fluid cytology and this may have resulted in an inflammatory response resulting in high ascitic fluid PMNs. 

Treatment of AL amyloidosis consists of autologous hematopoietic cell transplantation in patients who are transplant eligible, or with chemotherapy with melphalan, bortezomib, cyclophosphamide, and steroids who are transplant ineligible [13]. Prognosis varies considerably depending on the nature, number, and extent of organ involvement with median survival being as short as four to six months with advanced disease and multiorgan involvement [14].

## Conclusions

Amyloidosis should be suspected in patients if more than one organ system is involved and if laboratory, imaging, and clinical findings are not explained by a unifying diagnosis. Early diagnosis is important, as delayed diagnosis carries extremely poor outcomes despite the treatment of underlying etiology. Unfortunately, our patient had multi-organ involvement at the time of diagnosis, leading to mortality. Clinicians should be aware that although cirrhosis is the most common cause of ascites, other etiologies should be considered especially in patients with unusual presentations, inadequate response to traditional treatment, atypical ascitic fluid analysis, and those with multisystem involvement.

## References

[1] Monzawa S, Tsukamoto T, Omata K, Hosoda K, Araki T, Sugimura K (2002). A case with primary amyloidosis of the liver and spleen: radiologic findings. Eur J Radiol.

[2] Gertz MA, Kyle RA (1988). Hepatic amyloidosis (primary [AL], immunoglobulin light chain): the natural history in 80 patients. Am J Med.

[3] Urban BA, Fishman EK, Goldman SM, Scott WW Jr, Jones B, Humphrey RL, Hruban RH (1993). CT evaluation of amyloidosis: spectrum of disease. Radiographics.

[4] Chamarthi B, Dubrey SW, Cha K, Skinner M, Falk RH (1997). Features and prognosis of exertional syncope in light-chain associated AL cardiac amyloidosis. Am J Cardiol.

[5] Kim SH, Han JK, Lee KH (2003). Abdominal amyloidosis: spectrum of radiological findings. Clin Radiol.

[6] Merlini G, Dispenzieri A, Sanchorawala V, Schönland SO, Palladini G, Hawkins PN, Gertz MA (2018). Systemic immunoglobulin light chain amyloidosis. Nat Rev Dis Primers.

[7] Merlini G, Bellotti V (2003). Molecular mechanisms of amyloidosis. N Engl J Med.

[8] Zhao L, Tian Z, Fang Q (2016). Diagnostic accuracy of cardiovascular magnetic resonance for patients with suspected cardiac amyloidosis: a systematic review and meta-analysis. BMC Cardiovasc Disord.

[9] Mahmood S, Bridoux F, Venner CP (2015). Natural history and outcomes in localised immunoglobulin light-chain amyloidosis: a long-term observational study. Lancet Haematol.

[10] Park MA, Mueller PS, Kyle RA, Larson DR, Plevak MF, Gertz MA (2003). Primary (AL) hepatic amyloidosis: clinical features and natural history in 98 patients. Medicine.

[11] Levine RA (1962). Amyloid disease of the liver. Correlation of clinical, functional and morphologic features in forty-seven patients. Am J Med.

[12] Karoui S, Haddad W, Serghini M (2011). Peritoneal amyloidosis: Unusual localization of gastrointestinal amyloidosis. Clin J Gastroenterol.

[13] Kyle RA, Gertz MA, Greipp PR, Witzig TE, Lust JA, Lacy MQ, Therneau TM (1997). A trial of three regimens for primary amyloidosis: colchicine alone, melphalan and prednisone, and melphalan, prednisone, and colchicine. N Engl J Med.

[14] Kyle RA, Gertz MA, Greipp PR, Witzig TE, Lust JA, Lacy MQ, Therneau TM (1999). Long-term survival (10 years or more) in 30 patients with primary amyloidosis. Blood.

